# Evaluation of the interrater and intermethod agreement of the German multiparametric ultrasound criteria for the grading of internal carotid artery stenosis

**DOI:** 10.1007/s00234-020-02546-1

**Published:** 2020-09-18

**Authors:** Cindy Richter, Anna Weinreich, Simone Mucha, Dorothee Saur, Johann Otto Pelz

**Affiliations:** 1 Department of Neuroradiology, University Hospital Leipzig, Leipzig, Germany; 2 Epilepsy-Center Berlin-Brandenburg, Berlin, Germany; 3 Department of Neurology, University Hospital Leipzig, Liebigstrasse 20, 04103 Leipzig, Germany

**Keywords:** Ultrasound, Carotid artery disease, Interrater and intermethod agreement, Grading of internal carotid artery stenosis, DEGUM ultrasound criteria

## Abstract

**Purpose:**

The interdisciplinary German guidelines for the diagnosis and treatment of internal carotid artery stenosis (ICAS) recommend a multiparametric approach for the sonographic grading of extracranial ICAS. The aim of this study is to evaluate the interrater and intermethod agreement of this elaborated sonographic approach with different angiographic modalities.

**Methods:**

Patients with extracranial ICAS were examined twice with colour-coded duplex sonography (CDS) by two experienced vascular neurologists. Each of the ten criteria and the resulting stenotic value were assessed. Grading of ICAS based on the multiparametric ultrasound criteria was compared with different angiography modalities (magnetic resonance angiography (MRA), computed tomography angiography (CTA), digital subtraction angiography (DSA)).

**Results:**

Seventy-four consecutive patients with 91 extracranial ICAS were recruited from our stroke unit and neurovascular outpatient clinic. Interrater agreement for each single ultrasound criterion ranged from moderate to excellent (for the peak systolic velocity). Concerning the absolute stenotic value of ICAS, an excellent agreement between both ultrasound examiners with an ICC of 0.91 (range 0.87–0.94; *p* < 0.001) was found. In 96% of ICAS, the difference between the stenotic values was ≤ 10%. Intermethod agreements between CDS and DSA, CTA, and MRA were also good for both sonographers.

**Conclusion:**

Strictly adhering to the multiparametric “DEGUM ultrasound criteria”, we found an excellent interrater agreement and a good intermethod agreement compared with angiography for the sonographic grading of extracranial ICAS. Thus, multiparametric CDS is in particular suitable for the follow up of extracranial ICAS even when examinations are done by different sonographers.

## Introduction

Since patients with an at least moderate (≥ 50% distal diameter reduction percentage according to the North American Symptomatic Carotid Endarterectomy Trial (NASCET) [1]) symptomatic extracranial internal carotid artery stenosis (ICAS) harbour a substantially increased risk of early ischemic stroke recurrence after the index event [2], the early identification and correct grading of extracranial ICAS is of utmost importance in the work up of acute ischaemic stroke [3].

While imaging modalities for the detection and grading of extracranial ICAS directly visualise the stenosis by luminal sparing of the contrast agent as in computed tomography angiography (CTA), contrast-enhanced magnetic resonance angiography (CE-MRA), digital subtraction angiography (DSA) or three-dimensional ultrasound [4, 5], colour-coded duplex sonography (CDS) relies on haemodynamic changes caused by the stenosis. Although the grading of ICAS by CDS was repeatedly demonstrated to yield good agreements with the stenotic values measured by angiographic imaging modalities [6], CDS is still considered a subjective imaging modality which depends on the experience of the sonographer and technical equipment [7, 8]. The reason for this might be the lack of widely accepted ultrasound criteria; thus, ultrasound parameters and cut-off values for the sonographic detection and grading of ICAS differ between ultrasound labs [9]. Nevertheless, CDS of the extracranial carotid arteries is routinely performed in ischaemic stroke patients in the acute setting in the hospital but also in the outpatient care. In 2010, the German society for ultrasound in medicine (DEGUM) proposed the revised multiparametric “DEGUM ultrasound criteria” for grading ICAS [10]. However, prospective data about the interrater and intermethod agreements (compared with CTA, CE-MRA, DSA) are still missing. A recent study even questioned this elaborated approach of grading ICAS in particular in comparison with CTA [11].

The aim of this study was to prospectively assess the interrater agreement for the multiparametric sonographic grading of extracranial ICAS as a whole and for each single main and additional criterion, and to compare the stenotic values obtained by these multiparametric ultrasound criteria with the stenotic values measured by CTA, CE-MRA, and DSA.

## Methods and materials

### Study population

The study was approved by the local Ethics Committee of the Medical Faculty of the University of Leipzig (reference number 246/15-ek), and all participants gave their informed consent. Patients could participate in this study, if they revealed an at least low-grade extracranial ICAS in the CDS examination, which was routinely performed by a medical technical assistant. Exclusion criterion was a relevant intracranial stenosis ipsilateral to the extracranial ICAS that could affect accurate ultrasound-based grading. Intracranial carotid stenosis was identified either by angiography, if performed, or by transcranial CDS. For the latter, the discrepancy between the stenotic value of an extracranial low to moderate ICAS and the detection of collaterals and/or a compromised flow in the ipsilateral middle cerebral artery was indicative for a haemodynamically relevant and leading intracranial carotid artery stenosis. Angiography (CE-MRA, CTA, DSA) was not an integral part of this study and only performed if clinically indicated.

### Colour-coded duplex sonography

Patients were examined prospectively by two experienced and DEGUM-certified vascular neurologists (JP and AW) and with the same ultrasound system (Siemens X700, Siemens, Erlangen, Germany; Siemens Acuson 2000, Erlangen, Germany). At the time of the CDS examination, examiners were blinded to the results of the other examiner, of the medical technical assistant and of the results from other imaging modalities (angiography). Patients were lying in a supine position with the upper part of the body slightly elevated. The settings of the ultrasound systems were optimized for each patient with respect to gain, depth and focus.

Briefly, the sonographic grading according to the multiparametric DEGUM ultrasound criteria is based on a set of five main and five additional criteria. Morphological measurements (B-mode images and colour flow imaging) are the main criteria for low degrees of stenosis. Increasing peak-systolic and peak-diastolic velocities in the stenosis indicate an increasing luminal narrowing of the stenosis. The appearance of a collateral flow via either the anterior cerebral artery, the posterior communicating artery or the ophthalmic artery and a decreased poststenotic flow velocity proves a high degree stenosis (≥ 70% NASCET). Additional criteria like the extent of poststenotic flow disturbances, the confetti sign or the carotid ratio (peak systolic velocity of the internal to the common carotid artery) complement the grading (Table 1 [12]). Subsequently, each single main and additional criterion was assessed separately, and a representative image for each criterion was stored. At the end of the study, each examiner measured the stenotic value of the ICAS based on the stored images and according to the multiparametric DEGUM ultrasound criteria.

**Table 1:** Multiparametric “DEGUM ultrasound criteria” for the sonographic grading of internal carotid artery stenosis (modified from [9, 11]

Stenotic value (NASCET) in %	10	20–40	50	60	70	80	90	Occlusion
Main criteria								
1. B-mode image	+	+						
2. Colour duplex image	+	+	+	+	+	+	+	+
3. PSV intrastenotic (cm/s)			200	250	300	350–400	100–500	
4. PSV poststenotic (cm/s)					> 50	< 50	< 30	
5. Collateral flow (ophthalmic artery, ACA, PcomA)					+	+++	+	+
Additional criteria								
6. Pulsatility (CCA) or prestenotic resistancy index					+	+	+	+
7. Poststenotic flow disturbances			+	+	+	+	+	
8. EDV intrastenotic (cm/s)			< 100	< 100	> 100	> 100		
9. Confetti sign				+	+	+		
10. PSV carotid ratio (ICA / CCA)			≥ 2	≥ 2	≥ 4	≥ 4		

*NASCET* North American Symptomatic Carotid Endarterectomy Trial, *PSV* peaksystolic velocity, *ACA* anterior cerebral artery, *PcomA* posterior communicating artery, *CCA* common carotid artery, *EDV* enddiastolic velocity, *ICA* internal carotid artery, “+” means that the criterion is present or fulfilled

Since the accurate grading of the main criteria “B-mode image”, “Colour duplex image” and “Collateral flow” as well as of the additional criteria “Poststenotic flow disturbances” and the “Confetti sign” was subjective to a certain degree, we dichotomised them into present or absent and did not use a semiquantitative scale ranging from “(+)” to “+++” like in the original publications [10, 12]. The criterion Collateral flow was considered as present, if a retrograde flow was detected either in the ophthalmic artery, in the anterior cerebral artery or in the posterior communicating artery ipsilateral to the ICAS.

### CT-angiography

All CTA examinations were performed using a 128-section multidetector scanner (Ingenuity, Philips) with a slice thickness of 0.8 mm. Fifty millilitres of Imeron 400®, an iodine-based contrast agent (400 mg iodine per ml; Bracco Imaging Germany GmbH, Konstanz, Germany), was administered intravenously followed by a saline flush. Luminal diameters were measured by two neuroradiologists in thin-layer reconstructions via the software via Intellispace Portal preferably in the transversal plane and explicitly not in maximum-intensity projections. Quantification of ICA stenosis was then performed according to the NASCET criteria using the narrowest diameter of the stenotic lumen and the normal lumen of the distal ICA for calculation [1]. At the time of the measurements, both neuroradiologists were unaware of each other’s results.

### MR-angiography

Imaging was performed on a 3 Tesla magnetic resonance (MR) scanner (Siemens Trio, Siemens Healthcare, Erlangen, Germany) or on different 1.5 Tesla MR scanners (Achieva or Ingenia, Philips Healthcare, Best, Netherlands and Magnetom Symphony, Siemens Healthcare, Erlangen, Germany) in a clinical setup. The gadolinium-based contrast agent (Gadovist®, Bayer AG, Leverkusen, Germany) was injected intravenously. For an average-sized patient with a normal renal function, 10 ml of contrast agent was administered followed by a saline flush. For intracranial and extracranial CE-MRA, a coronally oriented 3D FLASH sequence was used, covering the intracranial and the cervical vessel from the aortic arch (voxel size 1.0 [3T] − 1.7 × 0.7 [3T] − 1.2 × 0.9 [3T] − 1.5 mm, scan time 44 s [54 s 3 T] per scan [4 repetitions], slices 64 [83 at 3 T], TR 3.23 ms [2.88 3 T], TE 1.2 [1.08 3 T], flip angle 25° [20° at 3 T]). Quantification of ICA stenosis was performed via Intellispace Portal reconstruction according to CTA evaluation.

### Diagnostic transarterial angiography

Diagnostic digital subtraction angiography (DSA) was performed by neuroradiologists using either a biplane Siemens system (Axiom Artis, Erlangen, Germany) or a monoplane GE system (Innova 4100; GE Healthcare, Waukesha, Wisc). Iopromid (60–120 ml, containing 300 mg iodine per ml) was used as the contrast agent. For catheter placement, a femoral artery approach was employed in which the tip of a 4-F or 5-F catheter (Tempo® Catheter, Cordis, Miami, FL) was guided from the right or left common femoral artery to the ascending aorta and positioned in the right and left common carotid arteries. After the selective catheterization, at least three different views (posteroanterior, lateral and 45° oblique) were obtained for all patients. Quantification of ICA stenosis was measured according to the NASCET criteria [1].

### Statistical analysis

Statistical analyses were performed with SPSS version 24.0 (IBM Corporation; New York, NY, USA). The interrater agreements for the stenotic values of ICAS and each velocity-based criterion of the DEGUM ultrasound criteria were visualised and described by a Bland and Altman analysis [13] and/or by the calculation of the intraclass correlation coefficient (ICC). The ICC estimates and their 95% confident intervals were calculated based on an absolute agreement and a 2-way mixed-effects model. The ICC values (ranging from 0 to 1) were interpreted as follows: excellent agreement ICC ≥ 0.90, good agreement ICC ≥ 0.75, moderate agreement 0.75 > ICC ≥ 0.50 and poor agreement ICC < 0.5 [14]. The ICC was also calculated to describe the intermethod agreements between CDS and CE-MRA, CTA and DSA for the stenotic values of ICAS.

Stepwise multivariate linear regression analyses were performed to explore the predictive impact of each ultrasound criterion for the total stenotic value of ICAS as assessed by DSA and by CTA. The stenotic value which was measured by DSA or CTA was the dependent variable, and the third to tenth ultrasound criteria were the independent variables. Since the first and the second criteria are particularly helpful for the assessment of low-grade stenosis and since most ICAS exhibited an aliasing phenomenon, i.e. the second criterion was present in most ICAS, these first two criteria were not included in the model.

In clinical practice, it is important to detect ICAS that may warrant revascularisation, i.e. to discriminate low-grade and moderate (stenotic value < 70% according to NASCET) from severe (stenotic value ≥ 70% according to NASCET) ICAS. Thus, logistic regression analyses were calculated with the stenotic value, which was measured by CTA and dichotomised into < 70% and ≥ 70% according to NASCET as the dependent variable and all but the first two criteria as the independent variables. Sensitivity and specificity for the detection of a severe (≥ 70% according to NASCET) ICAS were calculated for the multiparametric ultrasound criteria with the CTA as reference.

The correlation between a single criterion and the stenotic value measured by DSA was assessed with the Pearson correlation coefficient. A *p* value < 0.05 indicated statistical significance.

## Results

From February 2016 to November 2017, seventy-four consecutive patients (23 female, mean age 70 ± 12 years) were recruited from our stroke unit and neurovascular outpatient clinic. Fifty-seven patients had a unilateral ICAS, and 17 patients had bilateral ICAS. Two patients with two ICAS were examined by only one sonographer (resulting in a total of 72 patients with 89 ICAS that were sonographed twice). Both ultrasound examinations were performed at the same day in 70 (95%) patients.

**Fig. 1:**
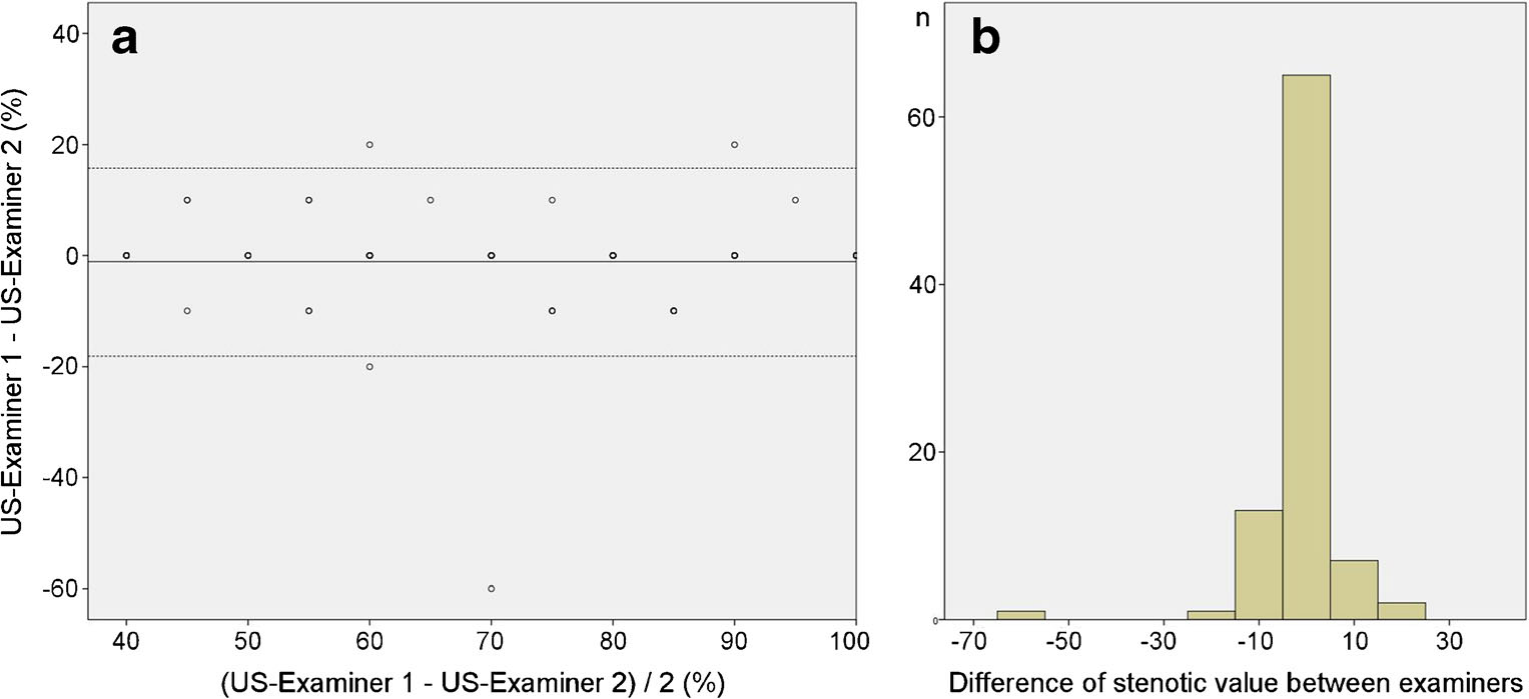
Interrater agreement of the sonographic grading of internal carotid artery stenosis. The interrater agreement for the grading of internal carotid artery stenosis between both sonographers when applying the multiparametric “DEGUM ultrasound criteria” is visualised by a Bland and Altman diagram **(a)**. In most cases, the difference of the total stenotic value was equal to/or less than 10% which again indicates a good agreement **(b)**

We found an excellent agreement between both ultrasound examiners concerning the stenotic value of ICAS with an ICC of 0.91 (confidence interval 0.87–0.94; *p* < 0.001) (Fig. 1a). In 85 of 89 (96%) ICAS, the difference between both ultrasound examiners for the stenotic values was ten or less percent (Fig. 1b). There was a discrepancy of 60% of the stenotic value between both examiners in one case which was due to a heavily calcified ICAS and a short and obese neck. The interrater agreements (ICC) for each single velocity–based main and additional criterion are shown in detail in Table 2.

**Table 2:** Interrater agreements for the single ultrasound criteria

	1. (0–40%)	2. (0–100%)	3. (50–90%)	4. (70–90%)	5. (70–100%)	6. (70–100%)	7. (50–90%)	8. (50–80%)	9. (60–80%)	10. (50–80%)	Stenotic value
*n*	17	88	60	39	56	56	62	53	42	51	89
ICC	–	–	0.90 (CI 0.84–0.94, *p* < 0.001)	0.72 (CI 0.52–0.84, *p* < 0.001)	–	0.60 (CI 0.38–0.75, *p* < 0.001)	–	0.81 ( CI 0.69–0.89, *p* < 0.001)	–	0.70 (CI 0.53–0.82, *p* < 0.001)	0.91 (CI 0.87–0.94; *p* < 0.001)
Agreement	11/17 (65%)	76/88 (86%)			46/56 (82%)		44/62 (71%)		25/42 (59%)		

Interrater agreements for each main (1 to 5) and each additional (6 to 10) criterion and the total stenotic value for colour-coded duplex sonography applying the multiparametric “DEGUM ultrasound criteria”. The semiquantitative criteria (1, 2, 5 – 7, 9) were dichotomised into present or absent and the percentage of agreement is given

*ICC* intraclass correlation coefficient, *CI* confidence interval

Intermethod agreements for the stenotic values of ICAS between CDS and the different angiographic modalities (CE-MRA, CTA, DSA) ranged from moderate to good with the limitation of a small number of MRA examinations (Table 3). Seven patients had an occlusion of the proximal internal carotid artery in the DSA that was diagnosed correctly in all cases by CDS. Comparing only the angiographic imaging modalities, there were good to excellent interrater agreements between both neuroradiologists for the stenotic value of ICAS measured by CE-MRA (ICC 0.92, confidence interval 0.77–0.98, *p* < 0.001; *n* = 13), by CTA (ICC 0.81, confidence interval 0.68–0.89, *p* < 0.001; *n* = 58), and by DSA (ICC 0.95, confidence interval 0.90–0.97, *p* < 0.001; *n* = 39).

**Table 3:** Intermethod agreements between stenotic values of ICAS measured by ultrasound and by contrast-enhanced angiography

	1st neuroradiologist			2nd neuroradiologist		
	MRA (*n* = 13)	CTA (*n* = 58)	DSA (*n* = 39)	MRA (*n* = 13)	CTA (*n* = 58)	DSA (*n* = 39)
1st CDS examiner	0.72 (CI 0.31–0.90, *p* = 0.002)	0.69 (CI 0.50–0.81, *p* < 0.001)	0.80 (CI 0.61–0.90, *p* < 0.001)	0.73 (CI 0.31–0.91, *p* = 0.002)	0.79 (CI 0.66–0.87, *p* < 0.001)	0.84 (CI 0.69–0.91, *p* < 0.001)
2nd CDS examiner	0.80 (CI 0.45–0.94, *p* < 0.001)	0.70 (CI 0.49–0.82, *p* < 0.001)	0.80 (CI 0.53–0.91, *p* < 0.001)	0.83 (CI 0.53–0.95, *p* < 0.001)	0.78 (CI 0.62–0.87, *p* < 0.001)	0.84 (CI 0.64–0.92, *p* < 0.001)

Intermethod agreement is described with the intraclass correlation coefficient (ICC)

*MRA* magnetic resonance angiography, *CTA* computed tomogram angiography, *DSA* digital subtraction angiography, *CI* confidence interval

**Fig. 2:**
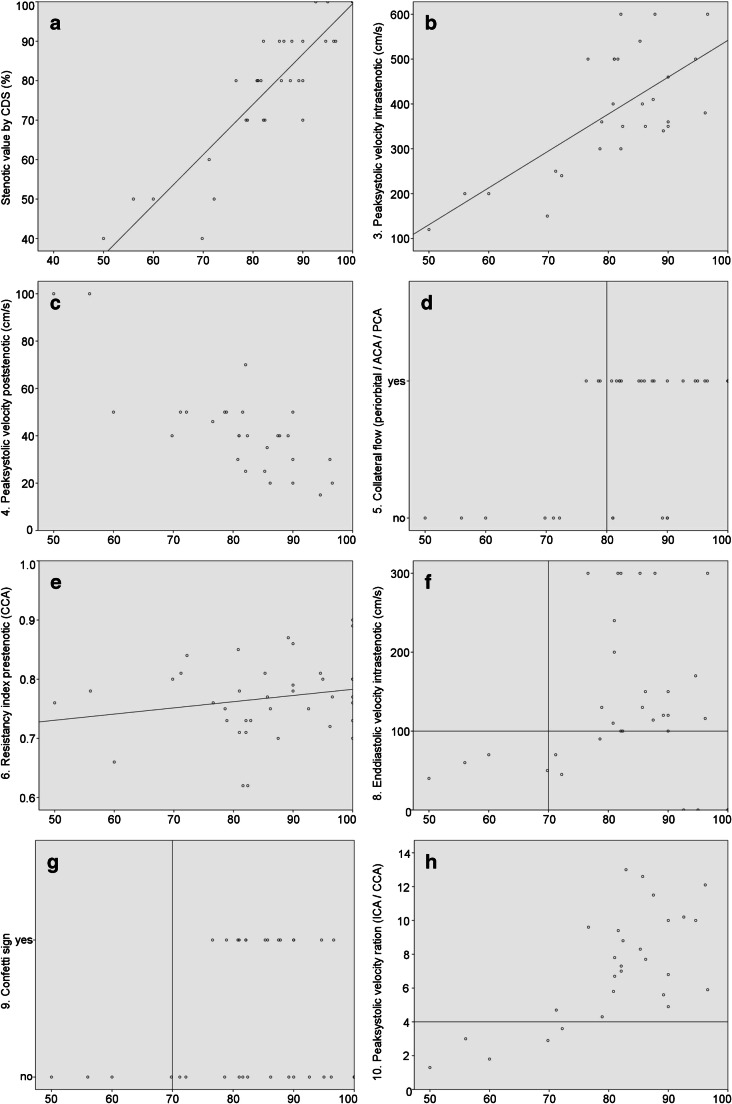
Correlation of the sonographic stenotic value and selected ultrasound criteria of the 2nd sonographer (y-axis) with the stenotic value assessed by digital subtraction angiography by the 2nd neuroradiologist (x-axis, in %). Overall, there was a good correlation between DSA and velocity-based criteria **(b**, **c**, **f**, **h)**. Both criteria that were dichotomised into present or absent (collateral flow **(d)** and confetti sign **(g)**) confirmed high-grade stenosis when positive but could not rule out a relevant stenosis when negative. The vertical and horizontal bars in **d**, **f**, **g**, **h** indicate the boundaries that are proposed to discriminate between a moderate and severe stenosis [9, 11]

Comparing the single sonographic main and additional criteria with the stenotic value of the ICAS assessed by DSA, most sonographic criteria demonstrated an at least good agreement or correlation (Fig. 2). The only exception was the prestenotic resistancy index (6^th^ criterion) which was unsuitable to discriminate a moderate from a severe ICAS (Fig. 2e).

Depending on which ultrasound examiner and which neuroradiologist measured the stenotic value of ICAS, stepwise multivariate linear regression analyses identified different combinations of sonographic criteria to best predict the stenotic value which was assessed via DSA (corrected *R*^2^ between 0.67 and 0.73) and CTA (corrected *R*^2^ between 0.53 and 0.63: Tables 4, 5, and 6). The peak systolic poststenotic velocity (4^th^ criterion) was present in seven of eight models (Table 4 and 5).

**Table 4:** Combinations of ultrasound criteria to best predict the stenotic value of internal carotid artery stenosis as measured by digital subtraction angiography

	1st neuroradiologist			2nd neuroradiologist		
1st CDS examiner	Collateral flow (5th) PSV carotid ratio (10th) Prestenotic resistancy index (6th)	*R*^2^ = 0.76	Corrected *R*^2^ = 0.73	Collateral flow (5th) PSV carotid ratio (10th) PSV poststenotic (4th)	R^2^ = 0.73	corrected R^2^ = 0.69
2nd CDS examiner	PSV poststenotic (4th) PSV intrastenotic (3rd) EDV intrastenotic (8th)	*R*^2^ = 0.70	Corrected *R*^2^ = 0.67	PSV poststenotic (4th) PSV intrastenotic (3rd) EDV intrastenotic (8th)	R^2^ = 0.75	corrected R^2^ = 0.72

A stepwise linear regression analysis was calculated for each combination of ultrasound examiner and neuroradiologist

*CDS* colour-coded duplex sonography, *PSV* peaksystolic velocity, *EDV* enddiastolic velocity

**Table 5:** Combinations of ultrasound criteria to best predict the stenotic value of internal carotid artery stenosis as measured by computed tomography angiography

	1st neuroradiologist			2nd neuroradiologist		
1st CDS examiner	EDV intrastenotic (8th) PSV poststenotic (4th)	*R*^2^ = 0.56	corrected R^2^ = 0.53	PSV carotid ratio (10th) PSV poststenotic (4^th^) Confetti sign (9th)	*R*^2^ = 0.65	Corrected *R*^2^ = 0.63
2nd CDS examiner	PSV poststenotic (4th) PSV intrastenotic (3rd)	*R*^2^ = 0.55	corrected R^2^ = 0.53	PSV poststenotic (4th) Collateral flow (5th)	*R*^2^ = 0.63	Corrected *R*^2^ = 0.62

A stepwise linear regression analysis was calculated for each combination of ultrasound examiner and neuroradiologist

*CDS* colour-coded duplex sonography, *PSV* peaksystolic velocity, *EDV* enddiastolic velocity

**Table 6:** Combinations of ultrasound criteria to best predict the stenotic value of internal carotid artery stenosis as measured by computed tomography angiography and dichotomised into low-grade to moderate (< 70%) versus severe (≥ 70%)

	1st neuroradiologist		2nd neuroradiologist	
1st CDS examiner	Poststenotic flow disturbances (7^th^)	Nagelkerkes *R*^2^ = 0.58	PSV intrastenotic (3^rd^) Prestenotic resistancy index (6^th^) EDV intrastenotic (8^th^) PSV carotid ratio (10^th^)	Nagelkerkes *R*^2^ = 0.76
2nd CDS examiner	No criterion was significant		Collateral flow (5^th^)	Nagelkerkes *R*^2^ = 0.70

A logistic regression analysis was calculated for each combination of ultrasound examiner and neuroradiologist

*CDS* colour-coded duplex sonography, *PSV* peaksystolic velocity, *EDV* enddiastolic velocity

Taking the CTA as the reference and considering the different combinations of ultrasound examiner and neuroradiologist, the sensitivity for the detection of a severe (≥ 70% according to NASCET) ICAS ranged between 0.66 and 0.81 with a specificity between 0.8 and 0.83.

**Fig. 3:**
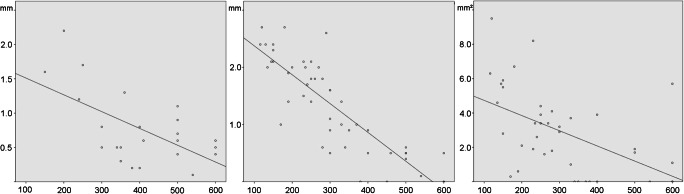
Correlation between intrastenotic diameter/cross-sectional area and peak systolic velocity of internal carotid artery stenosis. Correlation between the narrowest intrastenotic diameter (assessed by digital subtraction angiography **(a)** and CT angiography **(b)**), respectively, the narrowest intrastenotic cross-sectional area (assessed by CT angiography **(c)**) and the peak systolic intrastenotic velocity (*x*-axis, in cm/s) measured by colour-coded duplex sonography

The peak systolic velocity (3^rd^ criterion) correlated excellently inverse with the narrowest intrastenotic diameter in the CTA (Pearson, *r* = − 0.85), followed by the narrowest intrastenotic diameter in the DSA (Pearson, *r* = − 0.60) and the narrowest intrastenotic cross-sectional area in the CTA (Pearson, *r* = − 0.52) (Fig. 3).

## Discussion

The two main findings of this study were an excellent interrater agreement for the sonographic grading of ICAS and an overall good agreement between the stenotic values assessed by CDS in comparison with different angiographic imaging modalities when strictly adhering to the multiparametric DEGUM ultrasound criteria.

Traditionally, especially in Anglo-American countries, the peak systolic velocity (PSV) obtained at the maximum of the stenosis is the parameter of choice for the detection and grading of ICAS, although there are no worldwide accepted cut-off values for a moderate or severe ICAS. In addition, the PSV can be affected by several factors like the length of the ICAS, the collateral status, whether the ICAS is eccentric or not or by systemic parameters like the blood pressure or a polyglobulia [15]. Furthermore, PSV cut-offs for the detection of ICAS also depend on whether the ultrasound examiner favours to maximize sensitivity, specificity or accuracy for the detection of a moderate or a severe ICAS [16]. Taken these limitations, CDS is still regarded only as a screening examination and not the examination upon which treatment decisions should be based [8]. Noteworthy, the situation is different in Germany, where a multiparametric approach has been used for the sonographic detection and grading of ICAS since 1986 [15]. In 2010, these multiparametric criteria were revised and transferred to stenotic values according to NASCET and approved by all relevant German disciplines which are involved in the treatment of ICAS [10]. Finally, in 2012, this multiparametric approach was adopted with minor adjustments and recommended by the Neurosonology Research Group of the World Federation of Neurology [12].

In this study, when compared to DSA, the peak systolic velocity for severe ICAS ranged between 200 and 600 cm/s. Therefore, as was repeatedly shown before, the peak systolic velocity as the sole criterion is not sufficient for the grading or detection of (severe) ICAS [17]. Despite systemic factors like blood pressure, polyglobulia or the shape of the ICAS, this might in particular be due to the obligate correction of the insonation angle. Even small changes of the insonation angle lead to a considerable difference of the peak systolic velocity [17]. This might be overcome by new ultrasound techniques like vector flow imaging where the velocity measurement is independent of the insonation angle [18, 19]. Overall, no single CDS criterion showed a perfect agreement neither between both sonographers nor compared with DSA. However, stepwise linear regression analyses identified different combinations of sonographic criteria to best predict the stenotic value that was assessed via DSA or CTA. Therefore, the use of an elaborated multiparametric approach rather than some selected criteria might lead to redundancy in some cases, but the conclusive interpretation of the different sonographic criteria ensures an overall good intermethod and even excellent interrater agreement for the grading of ICAS. Consistently, an excellent interrater agreement for CDS was also described in a retrospective analysis of consecutive ultrasound examinations applying the DEGUM ultrasound criteria [20]. In this study as well as in our study, the interrater agreement for CDS was even better than the interrater agreement for CTA, although angiography is considered as the more objective imaging modality.

Because of its non-invasive character and excellent interrater agreement, CDS is particularly suitable for the follow up of ICAS [3]. An increase of the stenotic value of more than 10% between follow up examinations, even when the examinations are carried out by different sonographers, should be regarded as a real progression of ICAS over time. This might impact the treatment of those patients since the progression of the stenotic value is discussed as a risk factor for a subsequent ischaemic stroke [21, 22].

The assessment of the stenotic value of ICAS by CDS also showed a good agreement in comparison with the angiographic imaging modalities which strengthens the role of CDS in the diagnostic work up of ICAS [23]. Current guidelines still recommend that before undergoing carotid artery stenting or carotid endarterectomy, the stenotic value should be confirmed—preferably—by angiography or by a second ultrasound examination by an experienced sonographer [24]. As demonstrated in this study, CDS might be sufficient for the grading of ICAS when the sonographer is experienced, the examining conditions are good, and all of the ultrasound criteria can be assessed since a relevant discrepancy between two experienced examiners would be rare in these cases.

Moreover, in our study, all DSA-confirmed occlusions of the proximal internal carotid artery were correctly diagnosed by both sonographers. Using CT angiography, the differentiation between a near-occlusion, a pseudo-occlusion or a complete occlusion can be challenging [25–27].

In theory, the haemodynamical impact of the stenosis, i.e. the peak systolic velocity, should correlate best with the stenotic cross-sectional area (CSA) and not with the narrowest intrastenotic diameter [28]. However, we found the best correlation between the peak systolic velocity and the narrowest intrastenotic diameter in CTA and DSA. This might be due to difficulties in assessing the CSA exactly perpendicular to the centreline of the residual lumen, especially in severe ICAS, which might result in an overestimation of the stenotic CSA. Since the narrowest intrastenotic diameter and the narrowest intrastenotic CSA were measured in the same CTA, other systemic factors like differences in blood pressure between examinations were unlikely.

Our study has some limitations. At first, we included patients that had an at least low-grade (20 to 40% according to NACET) ICAS according to a preceding ultrasound examination by a medical technical assistant. Due to this preselection, our results cannot be interpreted in the context of the detection of ICAS. Secondly, a relevant intracranial ICAS was excluded by angiography in 64 of 74 (86.5%) patients. However, in the remaining 10 patients, it was excluded based on transcranial CDS which is not as accurate as angiography especially for the detection of severe stenosis of the distal intracranial internal carotid artery. Therefore, we cannot completely rule out the possibility that a few patients had a tandem stenosis of the internal carotid artery. Thirdly, since the decision for an angiography was a clinical one and not part of this study, there were only 39 pairs of CDS and DSA, which limits the good intermethod agreement. Moreover, since this was a monocentric study in a tertiary hospital, all DSA examinations were performed by very experienced neuroradiologists. This might also have impacted the good agreement between CDS and DSA. Contrary, in a multicentre study, Barlinn and colleagues reported only a moderate agreement and concluded that a confirmatory test is still needed for the grading of ICAS with CDS applying the multiparametric DEGUM ultrasound criteria [29]. In their Bland and Altman diagram, however, their agreement between CDS and DSA seemed to be better when only considering severe (≥ 70% NASCET) ICAS [29] like in our study. Fourthly, we did not assess parameters that are supposed to affect the haemodynamic effect of the ICAS like the blood viscosity (e.g. a polyglobulia or an increased erythrocyte sedimentation rate), the length of the stenosis and the systemic arterial blood pressure or the cardiac output. These parameters would not lead to differences in the sonographic grading of ICAS since most examinations were done within 1 day, however, they might lead to an under- or overestimation in particular of the velocity-based criteria and therefore impact the comparison with the angiographic modalities. Finally, only 13 ICAS were graded by CE-MRA which limits the intermethod comparison. Moreover, since (CE-MR) angiography was not an inherent part of this study, CE-MRA was performed on 1.5 and 3.0 Tesla MRI scanners. Thus, we cannot exclude that the different field strengths might have had an impact on the grading of ICAS.

In summary, we found an excellent interrater agreement and a good intermethod agreement compared with angiography for the sonographic grading of ICAS when strictly adhering to the multiparametric DEGUM ultrasound criteria. Thus, multiparametric CDS is in particular suitable for the follow up of ICAS even when examinations are done by different sonographers.

## References

[1] North American Symptomatic Carotid Endarterectomy Trial collaborators (1991). Beneficial effect of carotid endarterectomy in symptomatic patients with high-grade carotid stenosis. N Engl J Med.

[2] Ois A, Cuadrado-Godia E, Rodríguez-Campello A, Jimenez-Conde J, Roquer J (2009). High risk of early neurological recurrence in symptomatic carotid stenosis. Stroke.

[3] S3-Leitlinie zur Diagnostik, Therapie und Nachsorge der extracraniellen Carotisstenose. AWMF-Registernummer: 004-028. https://www.awmf.org/uploads/tx_szleitlinien/004-028l_extracranielle-Carotisstenose-Diagnostik-Therapie-Nachsorge_2020-02_03.pdf. Accessed 14.05.2020.

[4] Pelz JO, Weinreich A, Fritzsch D, Saur D (2015). Quantification of internal carotid artery stenosis with 3D ultrasound angiography. Ultraschall Med.

[5] Pelz JO, Weinreich A, Schob S, Saur D (2020). Multiparametric 3D contrast-enhanced ultrasound to assess internal carotid artery stenosis: a pilot study. J Neuroimaging.

[6] Wardlaw JM, Chappell FM, Best JJ, Wartolowska K, Berry E (2006). Non-invasive imaging compared with intra-arterial angiography in the diagnosis of symptomatic carotid stenosis: a meta-analysis. Lancet.

[7] Ranke C, Trappe H (1997). Blood flow velocity measurements for carotid stenosis estimation: interobserver variation and interequipment variability. Vasa.

[8] Latchaw RE, Alberts MJ, Lev MH, Connors JJ, Harbaugh RE, Higashida RT, Hobson R, Kidwell CS, Koroshetz WJ, Mathews V, Villablanca P, Warach S, Walters B (2009). Recommendations for imaging of acute ischemic stroke: a scientific statement from the American Heart Association. Stroke.

[9] Alexandrov AV, Vital D, Brodie DS, Hamilton P, Grotta JC (1997). Grading carotid stenosis with ultrasound: an interlaboratory comparison. Stroke.

[10] Arning C, Widder B, von Reutern G, Stiegler H, Görtler M (2010). Ultraschallkriterien zur Graduierung von Stenosen der A. carotis interna – Revision der DEGUM-Kriterien und Transfer in NASCET-Stenosierungsgrade. Ultraschall Med.

[11] Barlinn K, Floegel T, Kitzler HH, Kepplinger J, Siepmann T, Pallesen LP, Bodechtel U, Reichmann H, Alexandrov AV, Puetz V (2016). Multi-parametric ultrasound criteria for internal carotid artery disease-comparison with CT angiography. Neuroradiology.

[12] von Reutern GM, Goertler MW, Bornstein NM, Del Sette M, Evans DH, Hetzel A (2012). Grading carotid stenosis using ultrasonic methods. Stroke.

[13] Bland JM, Altman D (1986). Statistical methods for assessing agreement between two methods of clinical measurement. Lancet.

[14] Portney LG, Watkins MP (2000). Foundations of clinical research: applications to practice.

[15] Widder B, von Reutern GM, Neuerburg-Heusler D (1986). Morphologische und dopplersonographische Kriterien zur Bestimmung von Stenosierungsgraden an der A. carotis interna. Ultraschall Med.

[16] AbuRahma AF, Srivastava M, Stone PA, Mousa AY, Jain A, Dean LS (2011). Critical appraisal of the Carotid Duplex Consensus criteria in the diagnosis of carotid artery stenosis. J Vasc Surg.

[17] Widder B, Görtler M (2004). Doppler- und Duplexsonographie der hirnversorgenden Arterien.

[18] Goddi A, Bortolotto C, Fiorina I, Raciti MV, Fanizza M, Turpini E, Boffelli G, Calliada F (2017). High-frame rate vector flow imaging of the carotid bifurcation. Insights Imaging.

[19] Meyer P, Pelz JO (2018). Blood flow reversal from the external into the internal carotid artery - new insights into the hemodynamics at the carotid bifurcation. Brain Behav.

[20] Matz O, Nikoubashman O, Rajkumar P, Keuler A, Wiesmann M, Schulz J (2017). Grading of proximal internal carotid artery (ICA) stenosis by Doppler/duplex ultrasound (DUS) and computed tomographic angiography (CTA): correlation and interrater reliability in real-life practice. Acta Neurol Belg.

[21] Liam SH (2014). Progression rate and ipsilateral neurological events in asymptomatic carotid stenosis. Stroke.

[22] Kakkos SK, Nicolaides AN, Charalambous I, Thomas D, Giannopoulos A, Naylor AR, Geroulakos G, Abbott AL, Asymptomatic Carotid Stenosis and Risk of Stroke (ACSRS) Study Group (2014). Predictors and clinical significance of progression or regression of asymptomatic carotid stenosis. J Vasc Surg.

[23] Naylor AR, Ricco JB, de Borst GJ, Debus S, de Haro J, Halliday A, Hamilton G, Kakisis J, Kakkos S, Lepidi S, Markus HS, McCabe DJ, Roy J, Sillesen H, van den Berg JC, Vermassen F, ESVS Guidelines Committee, Kolh P, Chakfe N, Hinchliffe RJ, Koncar I, Lindholt JS, Vega de Ceniga M, Verzini F, ESVS Guideline Reviewers, Archie J, Bellmunt S, Chaudhuri A, Koelemay M, Lindahl AK, Padberg F, Venermo M (2018). Editor’s choice - management of atherosclerotic carotid and vertebral artery disease: 2017 clinical practice guidelines of the European Society for Vascular Surgery (ESVS). Eur J Vasc Endovasc Surg.

[24] Intercollegiate Stroke Working Party (2016). National clinical guideline for stroke.

[25] Görtler M, Niethammer R, Widder B (1994). Differentiating subtotal carotid artery stenoses from occlusions by colour-coded duplex sonography. J Neurol.

[26] Grossberg JA, Haussen DC, Cardoso FB, Rebello LC, Bouslama M, Anderson A (2017). Cervical carotid pseudo-occlusions and false dissections: intracranial occlusions masquerading as extracranial occlusions. Stroke.

[27] Kappelhof M, Marquering HA, Berkhemer OA, Borst J, van der Lugt A, van Zwam WH, Vos JA, Lycklama À Nijeholt G, Majoie CBLM, Emmer BJ, MR CLEAN Investigators (2018). Accuracy of CT angiography for differentiating pseudo-occlusion from true occlusion or high-grade stenosis of the extracranial ICA in acute ischemic stroke: a retrospective MR CLEAN substudy. Am J Neuroradiol.

[28] Dodds S (2002). The haemodynamics of asymmetric stenoses. Eur J Vasc Endovasc Surg.

[29] Barlinn K, Rickmann H, Kitzler H, Krogias C, Strohm H, Abramyuk A (2018). Validation of multiparametric ultrasonography criteria validation of multiparametric ultrasonography criteria with digital subtraction angiography in carotid artery disease: a prospective multicenter study. Ultraschall Med.

